# In Situ Constructing Highly Aligned Ribbon-like PHBV Lamellae in PBAT: Towards Strong, Ductile and High-Barrier PBAT/PHBV Films

**DOI:** 10.3390/ma18173947

**Published:** 2025-08-22

**Authors:** Yaqiao Wang, Jun Xu, Baohua Guo

**Affiliations:** Key Laboratory of Advanced Materials of Ministry of Education, Department of Chemical Engineering, Tsinghua University, Beijing 100084, China; yqwang2024@mail.tsinghua.edu.cn (Y.W.); jun-xu@mail.tsinghua.edu.cn (J.X.)

**Keywords:** PBAT, PHBV, in situ lamellarization, barrier property

## Abstract

This study presents a facile approach to fabricate PBAT/PHBV films with superior mechanical and barrier properties by in situ forming ribbon-like lamellae, achieving a PHBV platelet-reinforced PBAT films. The fabrication involves melt blending of PBAT and PHBV, where styrene–methyl methacrylate–glycidyl methacrylate copolymer as a multifunctional reactive compatibilizer (RC) regulates PHBV domain size by forming a branched/cross-linked PBAT-B-PHBV structure. The introduction of a compatibilizer into the PBAT/PHBV system can reduce domain size and improve interfacial adhesion, thereby elevating PBAT’s storage modulus and complex viscosity for optimized blow-molding processability. During blow-molding, biaxial stretching with rapid cooling transforms PHBV sea–island structures into well-aligned ribbon-like lamellae. Notably, when PHBV content is ≤30 wt.%, lamellae form in the PBAT matrix, significantly enhancing both mechanical and barrier properties. The addition of RC reduces the lateral dimensions of PHBV lamellae while increasing PHBV number density. The introduction of 0.2 wt.% RC optimizes lamellar dimensions and density to maximize permeation pathway tortuosity. Ultimately, the lamellae in the PBAT matrix yield remarkable property enhancements: yield strength increased by >600%, elastic modulus by >200%, and water vapor/oxygen transmission rate reduced by ~81% and ~85%, respectively.

## 1. Introduction

With the escalating global plastic pollution crisis, traditional petroleum-based plastic products are difficult to degrade in the natural environment, leading to ecological challenges such as soil compaction, water pollution and microplastic diffusion. According to statistics, more than 300 million tons of plastic waste are generated globally each year, among which film products such as packaging materials and other single-use products are important components. Their degradation cycle of up to 100 years seriously threatens environmental and biological safety [1]. Biodegradable films, made from materials such as polybutylene adipate terephthalate (PBAT), polyhydroxyalkanoate (PHA) and polylactic acid (PLA), can be broken down by microbial activity into water and carbon dioxide, thereby significantly reducing environmental residues in agricultural mulching, express packaging and other fields [2]. The development of biodegradable films is not only related to ecological safety but also a necessary technical path to achieve a circular economy [3].

PBAT (polybutylene adipate terephthalate) is an aliphatic–aromatic copolyester consisting of two different structural units. The aliphatic butylene adipate (BA) segments contribute to the material’s flexibility and biodegradability, while the aromatic butylene terephthalate (BT) segments provide rigidity and thermal stability [4,5]. Therefore, as an important raw material for biodegradable films, PBAT (polybutylene adipate terephthalate) features high ductility, good flexibility and film-forming ability, making it suitable for blow-molding processes. It is widely used in agricultural mulch films and packaging materials, and has been listed as an alternative to traditional plastics [6,7]. However, the soft-segment structure of PBAT also results in low elastic modulus and strength, as well as poor water vapor and oxygen barrier properties, which constrain its broader application in more demanding environments [8].

To address the issues of low mechanical strength and poor barrier properties in PBAT films caused by their soft-segment structure, some studies propose the following modification strategies: (1) Copolymerization modification: functional comonomers such as sodium 5-sulfoisophthalate [9] and 2,5-thiophenedicarboxylic acid [10] are used to synthesize PBAT-based copolyesters via copolymerization–condensation reactions. Compared to pure PBAT, these copolyesters exhibit superior mechanical and barrier properties. (2) Blend modification: rigid polymers (e.g., PLA, PBS) [11,12,13] or nano-inorganic materials (e.g., nanoclay (MMT) [14,15], cellulose nanocrystals (CNCs) [16,17]) are introduced into PBAT to enhance tensile strength and reduce oxygen permeability through phase behavior regulation and interfacial strengthening [18,19]. (3) Multilayer composite modification: the multilayer structure can construct efficient barrier layers for different permeates (such as oxygen, water vapor, aroma, etc.) through material combination optimization and interface design, and achieve the balance between strength and toughness through stress dispersion and functional layering [20]. Some studies constructed PBAT/PPC [21] and PBAT/PLA [22] multilayer composite films. As a result, the mechanical properties and barrier performance of the films are improved by utilizing the characteristics of different polymers and continuous multilayer structures. Although copolymerization modification and multilayer composite methods are able to enhance the barrier and mechanical properties of PBAT films, their processes are relatively complex and impose high requirements on equipment.

Fabrication of highly ordered lamellar architectures at the microscale has been shown to effectively enhance both the barrier and mechanical properties of films [23,24]. Some studies have demonstrated that 2 wt.% oriented graphene can reduce the water vapor permeability of TPU materials by 50% while significantly improving their mechanical performance. Concurrently, studies indicate that graphene with larger lateral dimensions exhibits a more pronounced effect on barrier property enhancement [25,26]. However, nanoscale fillers are constrained by limited lateral dimensions, and at high loadings, their high surface energy promotes self-aggregation into agglomerates. Elevated shear forces often fail to overcome the inter-particle aggregation tendency, thereby restricting the efficacy of nanoscale fillers.

In the process of polymer blending, it is feasible to induce the dispersed phase to form larger-lateral-dimension, aligned lamellar structures through stretching flow field. Polymer in situ fibrillation technology, which has attracted considerable attention in recent years, entails the application of unidirectional shear and tensile flow fields to molten blends [27,28,29]. This process induces instantaneous stretching of the dispersed phase into nanofiber architectures, which are subsequently solidified via rapid cooling to preserve the nanofiber morphology [30,31]. Notably, in situ fibrillation yields one-dimensional fibrous structures rather than two-dimensional lamellar configurations. Given the in situ fibrillation technology, modulating the flow field applied to the polymeric dispersed phase during in situ fibrillation processing to subject it to biaxial stretching may enable in situ formation of lamellar structures.

Thus, high-strength and high-barrier poly (3-hydroxybutyrate-co-3-hydroxyvalerate) (PHBV) [32,33] was selected for melt blending with PBAT to fabricate PBAT/PHBV blends, and a styrene–methyl methacrylate–glycidyl methacrylate copolymer as a multifunctional reactive compatibilizer was utilized to regulate the dispersed phase size of PHBV. During the blown film extrusion process, the blow-up ratio was set at the equipment’s upper limit of 4.5 to subject the polymer to maximal radial stretching. PBAT/PHBV/RC blends were subjected to biaxial stretching followed by rapid cooling, thereby enabling in situ formation of lamellar structures from the PHBV sea–island morphology. Consequently, PBAT/PHBV films were fabricated to exhibit a macroscopically single-layer structure with a microscopically multilayer architecture. This work further investigates the impact of PHBV lamellar structures on the mechanical and barrier properties of PBAT films. The in situ delamination technique enables a one-step fabrication of films with macroscopic single-layer/microscopic multilayer structures, with minimal requirements for processing techniques and equipment. These characteristics make the method highly promising for applications in the packaging, energy and transportation industries.

## 2. Materials and Methods

### 2.1. Materials

**Figure 1:**
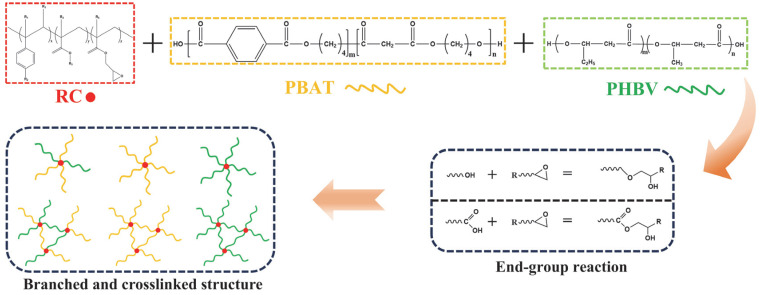
Possible molecular structure obtained in the reaction.

Polybutylene adipate co-terephthalate) (PBAT, Ecoworld) was supplied by Shanxi Jinhui Zhaolong High-tech Co., Ltd. (Taiyuan, China). The density is 1.23 g/cm^3^ and the melt flow index of the PBAT is 3–5 g/10 min (190 °C/2.16 kg). The copolymer of 3-hydroxybutyrate and 3-hydroxyvalerate (PHBV, ENMAT Y1000P) was produced by Ningbo Tianan Biomaterials Co., Ltd. (Ningbo, China). The content of 3-hydroxyvalerate is approximately 1 wt.%, and the density of PHBV is 1.25 g/cm^3^. The copolymer of styrene, methyl methacrylate and glycidyl methacrylate (ST37B) served as a reactive compatibilizer, produced by Anhui Xingbeida New Materials Technology Co., Ltd. (Tongling, China). The epoxy equivalent of ST37B ranges from 310 to 330 g/mol. Chemical structure of reactive compatibilizer is shown in Figure 1.

### 2.2. Sample Preparation

**Figure 2:**
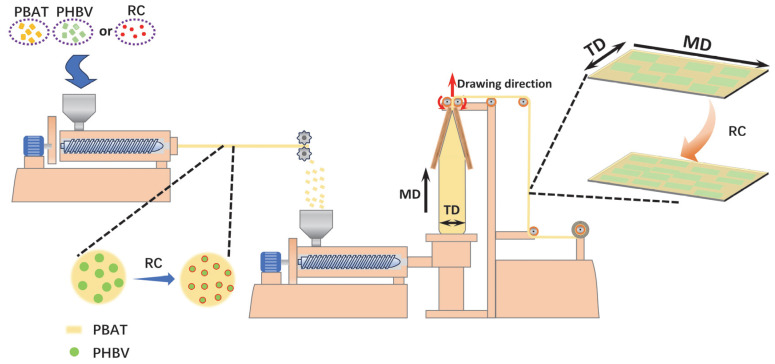
Schematic diagram of twin-screw extrusion and film blowing.

PBAT and PHBV were vacuum-dried at 80 °C for 8 h, while RC was vacuum-dried at 60 °C for 8 h to remove moisture in materials. The PBAT, PHBV and RC with different ratios were then fed into a twin-screw extruder (Lab Tech LTE20-40, Labtech Engineering Co., Ltd. Bangkok, Thailand) for melt blending or reactive blending. During melt extrusion, the temperature of the twin-screw extruder was set at 160–185 °C, and the screw rotation speed was 100 rpm. The prepared blend master batches were separately fed into a single-screw extruder (Lab Tech LE25-30/CV, Labtech Engineering Co., Ltd. Bangkok, Thailand), and the melt was delivered to a film-blowing machine (Lab Tech LF4110, Labtech Engineering Co., Ltd. Bangkok, Thailand) for film preparation. The temperature of the single-screw extruder during melt extrusion was maintained at 160–185 °C. The film-blowing machine was equipped with an annular die lip with an inner diameter of 10 cm and a die gap of 1 mm. To maximize the stretching of PHBV dispersed phase perpendicular to the drawing direction and increase its longitudinal dimension for forming lamellar structures, the blow-up ratio was set at the equipment’s upper limit of 4.5, and the draw ratio was set at 6.7, as shown in Figure 2. Eventually, a series of degradable blend films was prepared.

Blends were designated as PBAT, PBAT/PHBV10, PBAT/PHBV20, PBAT/PHBV30 and PBAT/PHBV40 corresponding to PHBV contents of 0 wt.%, 10 wt.%, 20 wt.%, 30 wt.% and 40 wt.%, respectively. Similarly, when PHBV content fixed at 30 wt.%, blends containing 0.1 wt.%, 0.2 wt.%, 0.3 wt.% and 0.4 wt.% of RC were designated as PBAT/PHBV/RC0.1, PBAT/PHBV/RC0.2, PBAT/PHBV/RC0.3 and PBAT/PHBV/RC0.4, respectively.

### 2.3. Infrared Spectroscopy Characterization

Fourier transform infrared spectroscopy (FTIR) was performed using a Nicolet IZ10 spectrometer (Thermo Fisher Scientific, Madison, WI, USA) to monitor chemical group transformations in RC and PBAT/PHBV during melt blending. Spectra were acquired from 3800 to 480 cm^−1^ at a resolution of 4 cm^−1^ with 32 co-added scans, ensuring adequate signal-to-noise ratio for detecting subtle chemical changes.

### 2.4. Gel Permeation Chromatography Characterization

All samples were dissolved in chloroform (3 mg/mL) for a minimum of 5 h to ensure complete solubilization. After filtration through a membrane molecular weight distribution (MWD) were characterized by GPC at 35 °C. The eluent flow rate was maintained at a constant 1.0 mL/min throughout the analysis.

### 2.5. Gel Content Characterization

The gel content of samples was determined following ASTM D2765-11. Samples were enclosed in a sealed 800-mesh cage and immersed in chloroform. The solvent was heated to 50 °C and maintained for 24 h, after which the cage was removed and rinsed with fresh chloroform for 10 min. Subsequently, the cage was dried in a vacuum oven at 80 °C for 24 h to eliminate residual solvent. The gel fraction was calculated using Equation (1):

(1) $$\mathrm{Gel}\;\mathrm{fraction}=\frac{W_{g}}{W_{0}}\times 100\%$$

where W_0_ is the sample weight before dissolution and W_g_ is the residual mass after dissolution.

### 2.6. Morphology Characterization

To characterize the morphological evolution of PHBV in blends, PBAT blends were cryogenically fractured after immersion in liquid nitrogen for 1 h. Fracture surfaces were sputter-coated with an ultrathin Au/Pd layer (≤20 nm) to enhance electrical conductivity, followed by SEM observation (FEI Quanta FEG250, Thermo Fisher Scientific, Hillsboro, OR, USA) of the dispersed phase morphology.

To characterize PHBV morphology in biodegradable blend films, PBAT films were cryofractured along the machine (MD) and transverse (TD) directions after liquid nitrogen immersion. Fracture surfaces were etched in a 40 °C NaOH/water/methanol mixture for 12 h to selectively remove PHBV domains, followed by ultrasonic cleaning in distilled water (10 min) and vacuum drying (6 h). Surfaces were sputter-coated with an ultrathin Au/Pd layer (≤20 nm) for conductivity, then imaged via the SEM. For quantitative analysis, Image Pro-Plus 6.0 statistically characterized PHBV domain sizes in blends and films, with size distributions plotted accordingly. The average size of the dispersed phase is determined by measuring at least 100 dispersed phase in the SEM images and calculating the mean value.

### 2.7. Rheological Property Characterization

To characterize the effect of PHBV dispersed phase on PBAT rheology and RC on PBAT/PHBV rheology, dynamic rheological properties were measured using a rotational rheometer (ARES Rheometer, TA Instruments, New Castle, IN, USA) at 185 °C over an angular frequency range of 0.1–100 rad/s. Strain was set at 1% to ensure measurements within the linear viscoelastic region. Tests were conducted using 25 mm diameter parallel plates with a 1 mm gap.

### 2.8. Thermal Property Characterization

Thermal properties were characterized using a differential scanning calorimeter (DSC Q100, TA Instruments, New Castle, IN, USA). Samples (8–10 mg) were heated to 190 °C and held for 3 min to eliminate thermal history, then cooled to −50 °C at 10 °C/min and held for 3 min to record the cooling crystallization curve. Subsequently, samples were reheated to 190 °C at 10 °C/min and held for 3 min to obtain the melting curve. Nitrogen was purged at 50 mL/min throughout the experiment to maintain an inert atmosphere.

### 2.9. Mechanical Performance Characterization

To investigate the effect of PHBV on the tensile properties of blend films, dumbbell-shaped specimens were cut along the MD and TD directions of the blend films. Tensile tests were performed using a universal testing machine (Instron 5900, Instron, Norwood, MA, USA) at a speed of 50 mm/min, and stress–strain curves were recorded. At least four replicates were conducted for each sample, with results averaged to ensure statistical significance.

To investigate the effect of PHBV on the tear resistance of blend films, right-angle tear specimens were prepared along the MD and TD directions of the blend films. Tear strength tests were performed using the universal testing machine at a speed of 200 mm/min, with right-angle tear strength values recorded. At least four replicates were conducted for each sample, and results were averaged to ensure statistical reliability.

To investigate the effect of PHBV lamellae on the puncture resistance of blend films, films were cut into 90 mm diameter circles and clamped between upper and lower annular fixtures with a 50 mm inner diameter. Puncture strength was measured using the universal testing machine, where a 1 mm diameter steel needle penetrated the film at a speed of 50 mm/min. Each sample was tested at least four times, and results were averaged to ensure statistical reliability.

### 2.10. Barrier Performance Characterization

Water vapor transmission rate (WVTR) was measured using a W303 vapor permeation tester (GBPI, Guangzhou, China) at 38 °C and 90% RH. Deionized water from a GN-RO-100 pure water system(Shanghai Youpu Industrial Co., Ltd. Shanghai, China) served as the permeant. At least three 80 mm diameter specimens per sample were tested, with results averaged for statistical validity. Oxygen transmission rate (OTR) was evaluated via differential pressure method using an N530G gas permeation analyzer (GBPI, Guangzhou, China) at 25 °C. High-purity oxygen (≥99.99%) was used as the test gas under 1 atm pressure.

## 3. Results and Discussion

### 3.1. Compatibilization of PBAT/PHBV Blends 

At elevated temperatures, epoxy groups in compatibilizer react with hydroxyl and carboxyl groups [34]. Figure 1 illustrates the possible reactions during melt blending. Below a specific threshold of RC content, only branched structures form; however, as RC content increases, molecular chains interconnected to form a cross-linked structure. The reaction of RC with PBAT and PHBV end groups during melt blending is a completely random process, potentially leading to the formation of branched and cross-linked PBAT, branched and cross-linked PHBV, branched PBAT-B-PHBV and cross-linked PBAT-B-PHBV, as shown in Figure 1.

**Figure 3:**
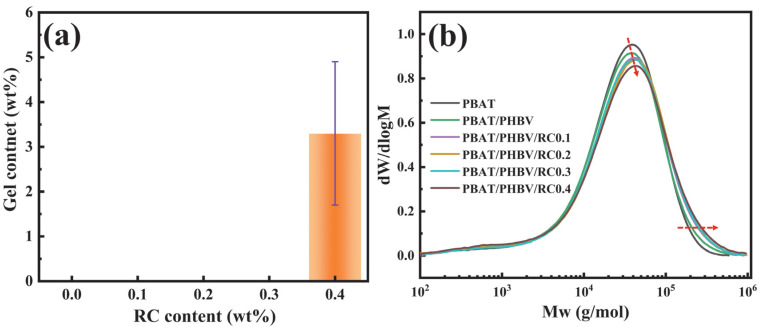
(**a**) gel content of PBAT/PHBV/RC blends; (**b**) molecular weight distribution of PBAT/PHBV/RC blends.

As shown in Figure 3a, when RC content exceeds 0.3 wt.%, the formation of chloroform-insoluble gels confirms the formation of a cross-linked structure. To investigate whether chain extension occurred at RC contents below 0.3 wt.%, GPC was employed for further analysis. After dissolving PBAT/PHBV/RC samples in chloroform, the molecular weight distribution curve was obtained by GPC testing, as shown in Figure 3b. For the partially compatible PBAT and PHBV in this study, the molecular weight distribution curves represent superimposed signals from both phases. The peak shift variations can reflect the compatibilizer’s influence on molecular architectures. Compared to pure PBAT, the introduction of PHBV results in the decrease in peak value while the increase in the high-mass tail. This stems from PHBV’s high molecular weight, which contributes to an increased fraction of high-molecular-weight components as observed in GPC analysis. With increasing RC content, the peak of molecular weight distribution curve exhibits a rightward shift. This further confirms the reaction of RC with PBAT or PHBV terminal hydroxyl and carboxyl groups to form structures with higher molecular weights [35].

### 3.2. Morphology of PBAT/PHBV and PBAT/PHBV/RC Blends

**Figure 4:**
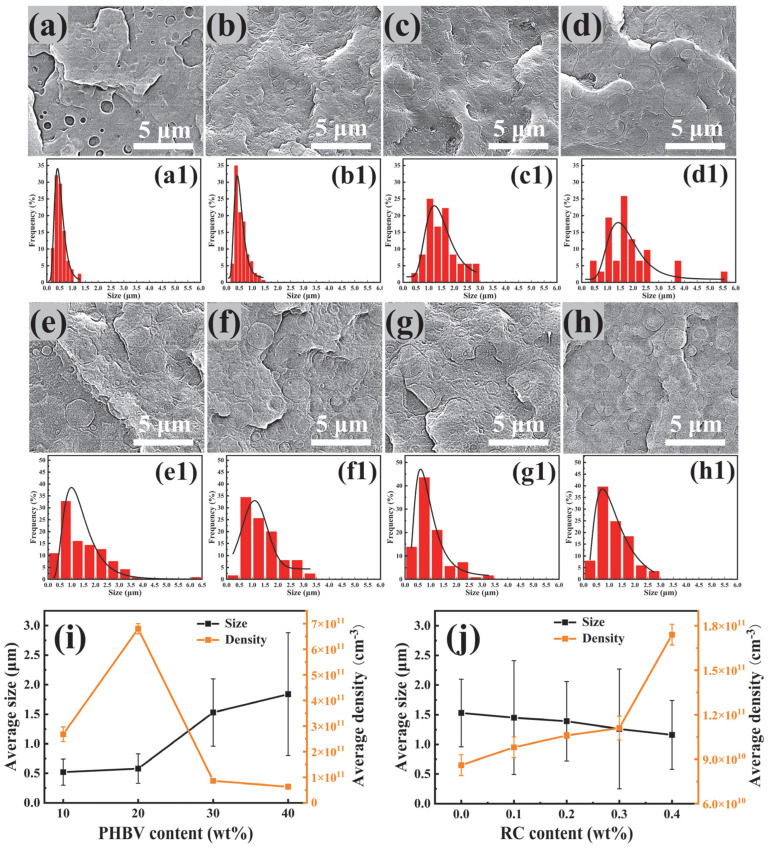
Morphology and cell size distribution of PBAT/PHBV and PBAT/PHBV/RC blends: (**a**,**a1**) PBAT/PHBV10, (**b**,**b1**) PBAT/PHBV20, (**c**,**c1**) PBAT/PHBV30, (**d**,**d1**) PBAT/PHBV40, (**e**,**e1**) PBAT/PHBV/RC0.1, (**f**,**f1**) PBAT/PHBV/RC0.2, (**g**,**g1**) PBAT/PHBV/RC0.3, (**h**,**h1**) PBAT/PHBV/RC0.4; (**i**) average size and density of the disperse phase with the increasing PHBV content; (**j**) average size and density of the disperse phase with the increasing RC content.

Figure 4 presents SEM images of fracture surfaces and dispersed phase size distributions for PBAT/PHBV and PBAT/PHBV/RC blends, illustrating PHBV dispersion in PBAT and the effect of compatibilizer content. As evident in Figure 4a–d,a1–d1, dispersed phase size increases with PHBV content, accompanied by broadening of the size distribution peak and a rightward shift on the *x*-axis. However, Figure 4i shows negligible size change below 20 wt.% PHBV, with significant growth above this threshold. Concurrently, dispersed phase density exhibits a non-monotonic trend, increasing initially then decreasing with rising PHBV content. This behavior stems from: at low loadings, large inter-particle distances reduce collision frequencies, suppressing coalescence. Thus, size remains stable despite increasing PHBV content, while density rises sharply. The density decline at high loadings reflects coalescence-driven aggregation. These findings indicate a PHBV dispersion concentration threshold in 20–30 wt.% under the experimental processing conditions.

Figure 4e–h,4e1–h1,j show that at 30 wt.% PHBV, increasing RC loading reduces dispersed phase size and increases phase density with rising RC content. As illustrated in Figure 2, RC epoxy groups chemically react with PBAT/PHBV end groups at elevated temperatures, potentially forming branched or cross-linked PBAT-B-PHBV structures. The branched or cross-linked PBAT-B-PHBV locate on the PBAT/PHBV interface, lowering interfacial tension and thus reducing dispersed phase size.

### 3.3. Rheological Behavior of PBAT/PHBV and PBAT/PHBV/RC Blends

**Figure 5:**
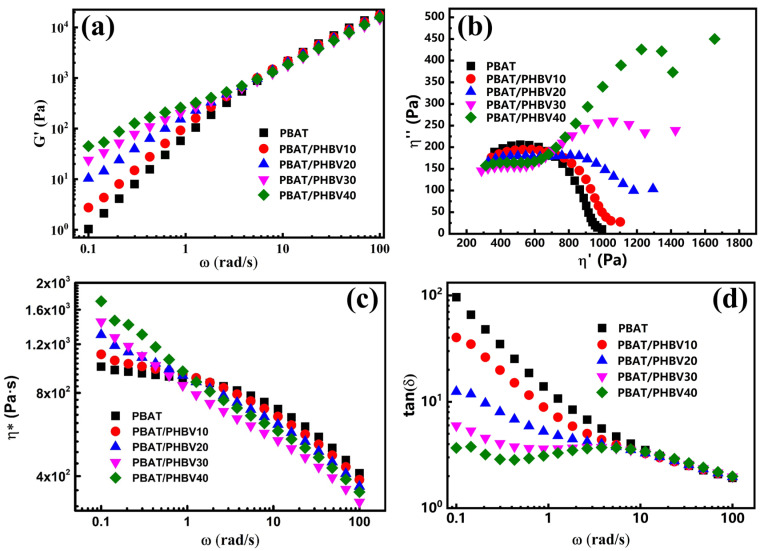
Dynamic rheological properties of PBAT/PHBV blends: (**a**) storage modulus, (**b**) Cole–Cole curves, (**c**) complex viscosity, (**d**) loss angle tangent.

The rheological behavior of polymers is pivotal for blow molding, directly impacting film quality, production efficiency and process stability. Figure 5a–d shows the storage modulus (G’), Cole–Cole curves, complex viscosity (η*) and loss tangent (tanδ) of PBAT/PHBV blends across angular frequencies (ω). As Figure 5a shows, G’ of the blend melt increases with PHBV content. The Cole–Cole plots in Figure 5b display pure PBAT has a single semicircular arc, indicating one relaxation mechanism. PBAT/PHBV blends begin to exhibit two distinct arcs with deviation from a semicircle, indicating the occurrence of phase separation or interfacial relaxation in the blend. The effects of interfacial interactions in the sea–island structure and viscosity contrast, collectively suppress the relative molecular chain motions in the molten blend, thereby enhancing elastic response of the system [36]. Figure 5c shows neat PBAT displays a Newtonian plateau at low frequencies. With higher PHBV loading, the blend melt’s low-frequency η* increases, and the Newtonian plateau vanishes within the testing frequency range. Concurrently, η* drops sharply with increasing frequency, indicating pronounced shear thinning. This indicates that PHBV simultaneously increases the complex viscosity of the blends. All samples show decreasing tanδ with ω, typical of viscoelastic liquids [37]. The tanδ curve of PBAT/PHBV blends declines with PHBV content, showing PHBV contributes more to elastic than viscous response. Enhanced melt elasticity and viscosity mitigate blow molding issues—such as melt fracture or sagging—preventing bubble collapse, thickness variation and film rupture.

**Figure 6:**
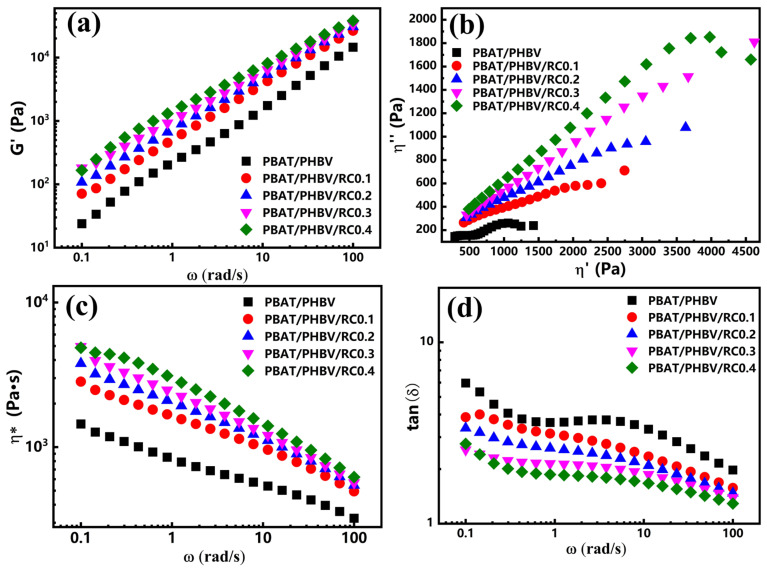
Dynamic rheological properties of PBAT/PHBV/RC blends: (**a**) storage modulus, (**b**) Cole–Cole curves, (**c**) complex viscosity, (**d**) loss angle tangent.

Figure 6a–d depicts the rheological properties of PBAT/PHBV/RC blends containing 30 wt.% PHBV across the frequency spectrum. As illustrated in Figure 6a, G’ of the blend melt increases with the addition of RC content, where the low-frequency G’ improves by approximately one order of magnitude when RC is incorporated into the PBAT/PHBV matrix. Notably, RC incorporation also significantly elevates high-frequency G’. This behavior is mainly attributed to branched structures formed by multifunctional reactive compatibilizers, which exacerbate chain entanglement and prolong relaxation times. Figure 6b presents the Cole–Cole plots of PBAT/PHBV/RC blends, where the progressive enlargement of the arc radius with increasing RC content provides additional evidence for RC’s role in enhancing the melt relaxation time of the blends. Additionally, increasing RC content reduces PHBV dispersed phase size and increases phase density, thereby expanding the interphase area. Branched PBAT-B-PHBV at the interface restricts PBAT chain mobility, representing another reason for G’ enhancement [38]. Figure 6c reveals that η* increases across all frequencies with RC content, due to intensified chain entanglement and interfacial effects hindering polymer chain migration. Figure 6d demonstrates that tanδ decreases gradually with RC content throughout the frequency range, indicating that RC-induced entanglement and interfacial effects predominantly enhance the elastic response. These findings confirm that RC compatibilization significantly improves melt elasticity, thereby maintaining bubble stability during blow molding operations.

### 3.4. Crystallization Behavior of PBAT/PHBV and PBAT/PHBV/RC Blends

**Figure 7:**
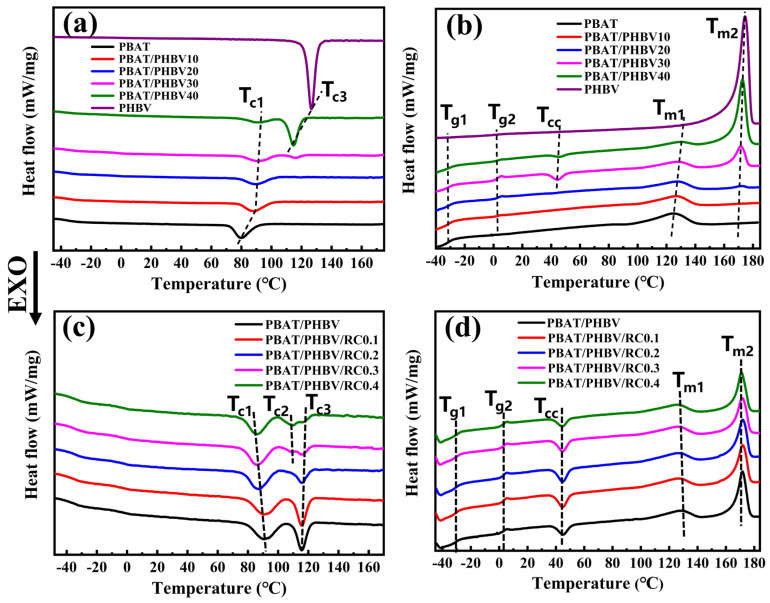
(**a**) Cooling and (**b**) heating graphs of PBAT/PHBV blends at a rate of 10 °C/min; (**c**) cooling and (**d**) heating graphs of PBAT/PHBV/RC blends at a rate of 10 °C/min.

Figure 7a,b illustrates the non-isothermal crystallization and melting curves of PBAT/PHBV blends. As Figure 7a illustrates, increasing PHBV content from 0 to 40 wt.% elevates PBAT’s crystallization temperature (T_c_) from 79.9 °C to 91.3 °C. A PHBV crystallization peak emerges at ~114 °C on DSC cooling curves with higher PHBV loadings. The glass transition temperatures of PBAT (T_g1_) and PHBV (T_g1_) remain unaltered in the blends, indicating poor miscibility between the phases. Owing to PHBV’s higher crystallization temperature than PBAT, it preferentially crystallizes during cooling, forming phase-separated structures. Interfacial regions act as nucleation sites, reducing PBAT’s crystallization activation energy and inducing heterogeneous nucleation, thereby enhancing T_c_ [39,40]. Figure 7b shows that PHBV introduction increases PBAT’s melting temperature (Tm) from 124.5 °C to 130.0 °C, while PHBV’s Tm stays stable at ~172 °C irrespective of content. This suggests interfacial nucleation not only boosts T_c_ but also promotes more perfect PBAT crystallization. Table 1 indicates PBAT’s melting enthalpy decreases with increasing PHBV content, attributed to both reduced PBAT concentration and hindered crystal growth by densely distributed PHBV domains [41].

Figure 7c,d depicts the non-isothermal crystallization and melting curves of PBAT/PHBV/RC blends. As Figure 7c illustrates, increasing RC content from 0 to 0.4 wt.% reduces PBAT’s crystallization temperature from 90.8 °C to 86.2 °C. This behavior arises from the formation of branched and cross-linked structures during reactive blending, which reduces chain mobility to impede the formation of critical-sized nuclei, thus lowering T_c_. When RC content exceeds 0.2 wt.%, a new PHBV crystallization peak emerges at 109 °C, which arises from the enhanced chain entanglement caused by branched and cross-linked structures formed during reactive blending. This reduces the driving force for molecular chains to pack into crystalline lattices, leading to a significant decrease in the crystallization temperature of partial PHBV and the formation of two crystallization peaks [42]. Table 1 shows that RC incorporation decreases PBAT’s melting temperature and slightly reduces melting enthalpy, indicating that branched structures not only impede chain diffusion into crystals but also diminish PBAT crystallite perfection.

### 3.5. Morphology of PBAT/PHBV and PBAT/PHBV/RC Films 

**Figure 8:**
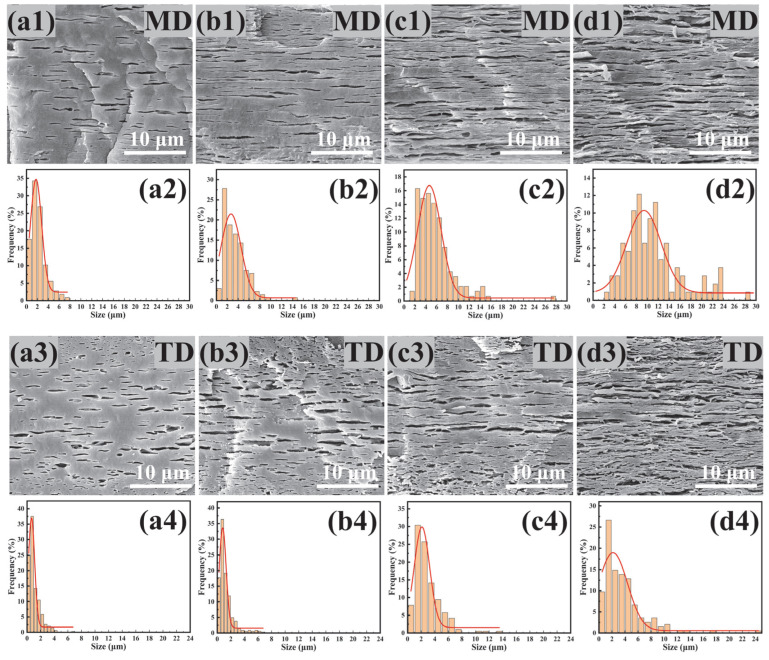
Sectional morphology of PBAT/PHBV thin films in MD (**a1**–**d1**) and TD (**a3**–**d3**) direction; size distribution of lamellar PHBV after etching away PHBV in MD (**a2**–**d2**) and TD (**a4**–**d4**) direction; (**a1**–**a4**) PBAT/PHBV10, (**b1**–**b4**) PBAT/PHBV20, (**c1**–**c4**) PBAT/PHBV30, (**d1**–**d4**) PBAT/PHBV40.

**Figure 9:**
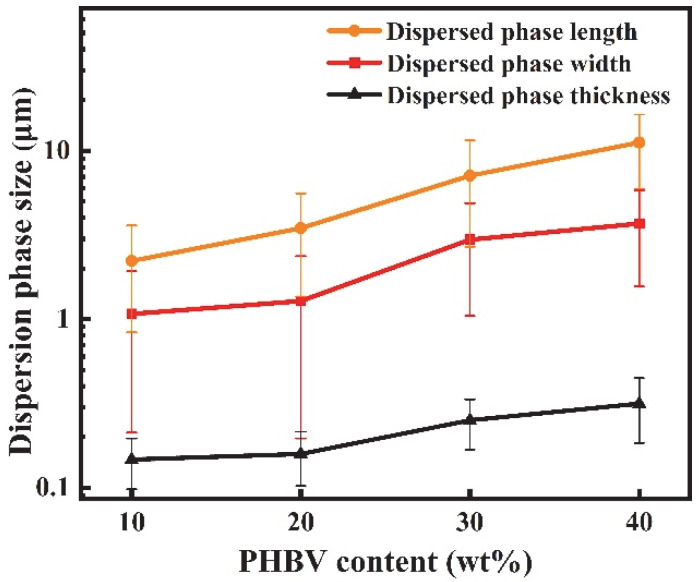
The change in lamellar PHBV size with the content of PHBV.

Figure 8 shows the cross-sectional morphology of PBAT/PHBV blend films after PHBV etching in NaOH solution. During the blown film extrusion, the blow-up ratio was set at the equipment’s upper limit of 4.5 to subject the polymer to maximal radial stretching. Therefore, PHBV spherical dispersed phases transform into lamellar structures under biaxial extension. Figure 8 illustrates that increasing PHBV content drives the growth of PHBV lamellae in machine, transverse and thickness directions. The length size distribution expands from 0.5–7.1 μm to 2.6–28.8 μm, while the width size distribution increases from 0.5–3.5 μm to 0.5–24.6 μm. Figure 9 shows the average length size of PHBV lamellae increases from 2.2 μm to 11.2 μm, with the average width size rising from 1.1 μm to 3.7 μm—evidence of stronger stretching in MD compared to TD during blow molding. Consequently, the PHBV sea–island structure evolves into ribbon-like lamellae under biaxial tension.

**Figure 10:**
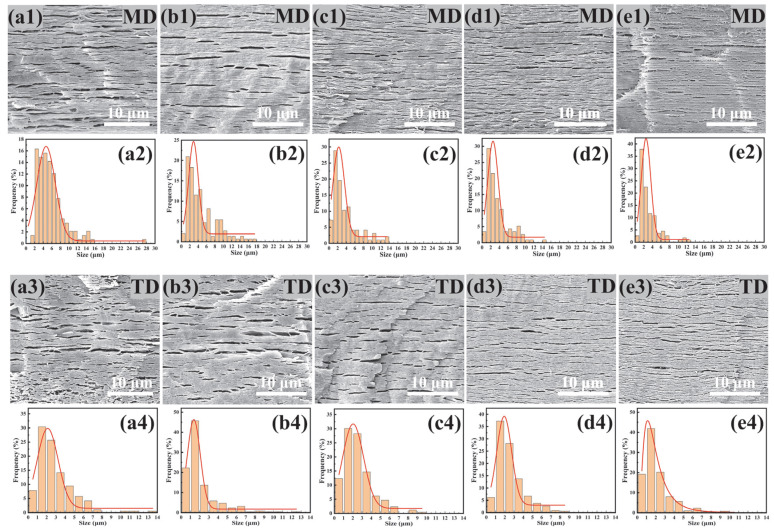
Sectional morphology of PBAT/PHBV/RC thin films in MD (**a1**–**e1**) and TD (**a3**–**e3**) direction; size distribution of lamellar PHBV after etching away PHBV in MD (**a2**–**e2**) and TD (**a4**–**e4**) direction; (**a1**–**a4**) PBAT/PHBV, (**b1**–**b4**) PBAT/PHBV/RC0.1, (**c1**–**c4**) PBAT/PHBV/RC0.2, (**d1**–**d4**) PBAT/PHBV/RC0.3, (**e1**–**e4**) PBAT/PHBV/RC0.4.

**Figure 11:**
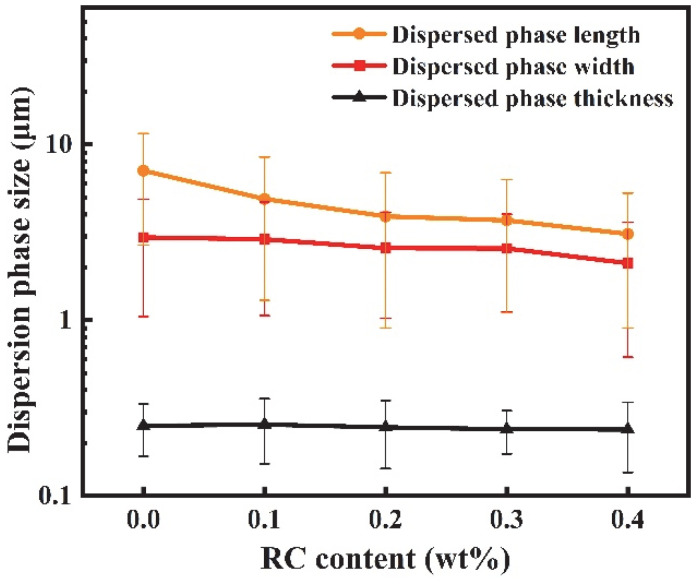
The change in lamellar PHBV size with the content of RC.

Figure 10 displays the cross-sectional morphology of PBAT/PHBV/RC blend films after PHBV etching in NaOH solution. SEM micrographs reveal that increasing RC content induces denser distributions of PHBV lamellae. Figure 11 shows that the average length size of PHBV lamellae decreases from 7.1 μm to 3.1 μm, and the average width size drops from 2.9 μm to 2.1 μm with the increase in RC content. As previously established, RC acts as a compatibilizer to enhance PBAT/PHBV miscibility, thereby reducing PHBV phase size and increasing phase density as shown in Figure 4i. Consequently, biaxial stretching during blow molding generates smaller-sized and higher-density PHBV lamellar structures with refined morphology. The incorporation of RC induces morphological alterations in PHBV lamellae, which increases the interfacial area and enhances interfacial compatibility to promote efficient stress transfer. However, the reduction in lamellar size and the increment in lamellar density exhibit antagonistic effects on the elongation of barrier pathways, which will be investigated in the following section.

### 3.6. Mechanical Property of PBAT/PHBV and PBAT/PHBV/RC Blend Films

**Figure 12:**
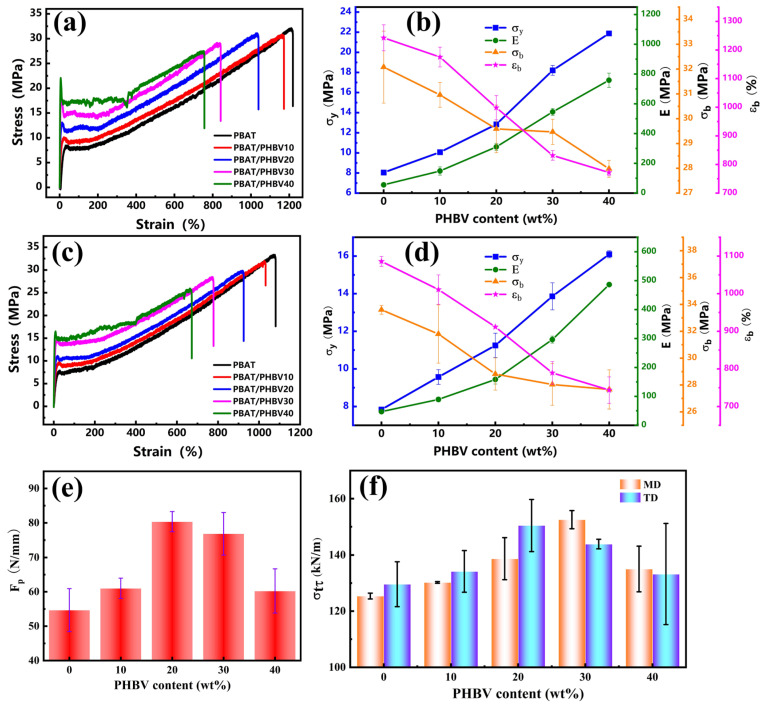
Mechanical properties of PBAT/PHBV: (**a**,**b**) stress–strain curves and tensile property parameters of PBAT/PHBV in MD; (**c**,**d**) stress–strain curves and tensile property parameters of PBAT/PHBV in TD; (**e**) the puncture properties of PBAT/PHBV films; (**f**) the tear properties of PBAT/PHBV films.

Figure 12a,c presents the tensile curves of PBAT/PHBV blend films in MD and TD directions, respectively. Figure 12b,d illustrates the corresponding tensile parameters as a function of PHBV content in MD and TD directions. In both MD and TD directions, increasing PHBV content elevates yield strength (σ_y_) and elastic modulus (E), while reducing tensile strength at break (σ_b_) and elongation at break (ε_b_). Compared to neat PBAT, the cold-drawing plateau stress in PBAT/PHBV films surges by ~100%. As a random copolymer, PBAT undergoes plastic deformation via molecular chain extension and orientation during stretching, forming necking or local yielding zones. PHBV—a brittle material with high strength and modulus—acts as a reinforcing phase when tensile stress transfers to PHBV lamellae. Rigid PHBV lamellae impede PBAT chain slippage, thereby boosting σ_y_ and E with higher PHBV loadings. However, poor PBAT/PHBV compatibility induces interfacial debonding, causing σ_b_ to decrease [43,44].

Figure 12e illustrates the puncture strength of PBAT/PHBV blend films as a function of PHBV content, exhibiting a non-monotonic trend with increasing PHBV. At PHBV contents of 20–30 wt.%, the puncture strength of PBAT/PHBV blend films increases by ~60% compared to neat PBAT, then declines at higher loadings. This behavior originates from the balance between PHBV-mediated crack resistance and stress concentration induced by excessive lamellar aggregation. Puncturing induces local high-stress gradients, triggering rapid crack initiation and propagation via shearing and tearing, thereby culminating in film failure. Rigid PHBV lamellae compel crack deflection or branching, increasing fracture surface area and energy dissipation to delay puncture failure [45]. However, Excess PHBV will shift the blend’s behavior from ductile to brittle, with stiffness and brittleness becoming pronounced. This severely reduces ductility, especially during puncture-induced localized large deformation. The material fails to absorb puncture energy through plastic deformation, weakening its overall puncture resistance.

Figure 12f shows the tear strength of PBAT/PHBV blend films in MD and TD directions as a function of PHBV content, both also exhibiting non-monotonic trends with increasing PHBV. Notably, the tear strength reaches its maximum value at 30 wt.% PHBV in MD direction, while it peaks at 20 wt.% PHBV in TD direction. As previously discussed, the PHBV lamellae exhibit parallel alignment in the film, displaying anisotropic dimensional distribution where the longer axes preferentially orient along MD and shorter axes along TD. Tearing along MD direction—where PHBV lamellae are parallel to the tear direction—causes cracks to propagate along lamellar interfaces or deflect around lamellae, extending the crack path and dissipating energy. Tearing along TD direction—where lamellar alignment is perpendicular to the tear direction—creates physical barriers that hinder crack propagation. However, excessive PHBV reduces lamellar space, triggering brittle fracture that supplants ductile tearing mechanisms, thus decreasing tear performance. This indicates that multilayered, large-sized lamellar structures aligned with the tear direction enhance tear resistance efficiency.

Given the optimal balance of mechanical strength and toughness at 30 wt.% PHBV, this study selects this content to introduce a reactive compatibilizer, further investigating the effect of PHBV dispersed phase morphology on film properties at the same loading.

**Figure 13:**
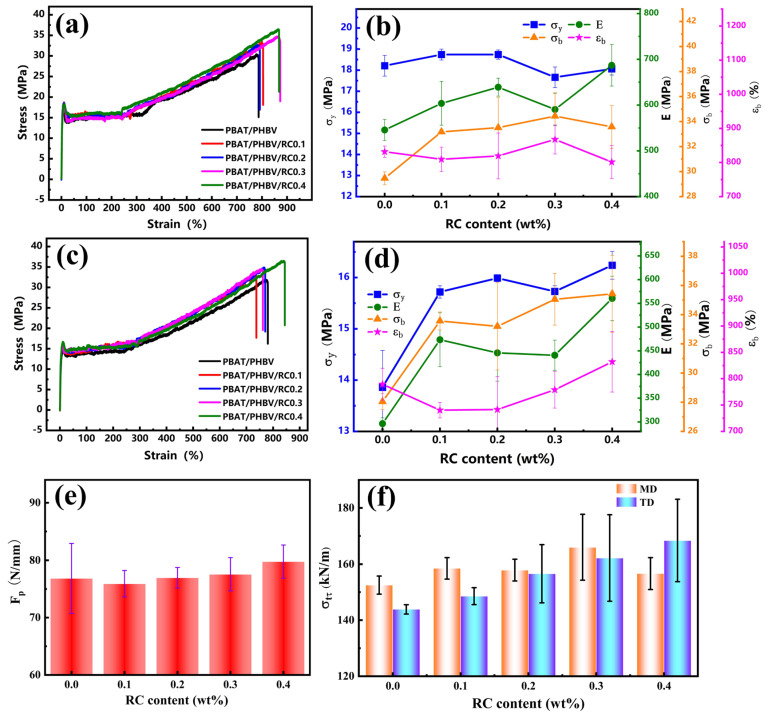
Mechanical properties of PBAT/PHBV/RC: (**a**,**b**) stress–strain curves and tensile property parameters of PBAT/PHBV/RC in MD; (**c**,**d**) stress–strain curves and tensile property parameters of PBAT/PHBV/RC in TD; (**e**) the puncture properties of PBAT/PHBV/RC films; (**f**) the tear properties of PBAT/PHBV/RC films.

Figure 13a,c presents the tensile curves of PBAT/PHBV/RC blend films with 30 wt.% PHBV addition in MD and TD directions, respectively. Figure 13b,d shows the variations of tensile parameters with RC content for PBAT/PHBV/RC blend films in MD and TD directions. In both directions, σ_b_ and ε_b_ of the PBAT/PHBV/RC blend films increase with increasing RC content. RC reacts with the end groups of PBAT and PHBV to form branched structures, which enhances physical entanglement between molecular chains and improves tensile strength and elongation. Additionally, the branched PBAT-B-PHBV strengthen interfacial adhesion, reduce interfacial defects and enhance stress transfer efficiency, thus boosting tensile properties. The as-prepared PBAT/PHBV/RC blend films exhibit higher tensile strength at break than pure PBAT films, with slightly lower elongation at break. Notably, their σ_y_ and E are increased by more than 600% and 200% compared to pure PBAT films. 

Figure 13e depicts the correlation between the puncture strength of PBAT/PHBV/RC films and RC loading. The puncture strength of the films exhibits a gradual increment with increasing RC content. As previously elaborated, the elongation of polymer chains augments inter-chain physical entanglement, while the formation of branched architectures reinforces interfacial adhesion and increases interfacial areas. Consequently, these synergistic effects contribute to the enhanced puncture resistance of PBAT/PHBV/RC films.

Figure 13f shows the relationship between the tear strength of PBAT/PHBV/RC films in MD and TD directions as the increase in RC content. The tear strength of PBAT/PHBV/RC blend films in both MD and TD directions gradually increases with the increment of PHBV content. Similarly, this can be attributed to the enhanced physical entanglement of polymer chains and improved interfacial compatibility, which delay crack tip propagation and reduce local stress concentration caused by interfacial debonding. In summary, the incorporation of RC modifies the molecular structure and disperse phase morphology of the blend, leading to a comprehensive enhancement in the mechanical properties of PBAT/PHBV films.

### 3.7. Barrier Performance of PBAT/PHBV and PBAT/PHBV/RC Blend Films

**Figure 14:**
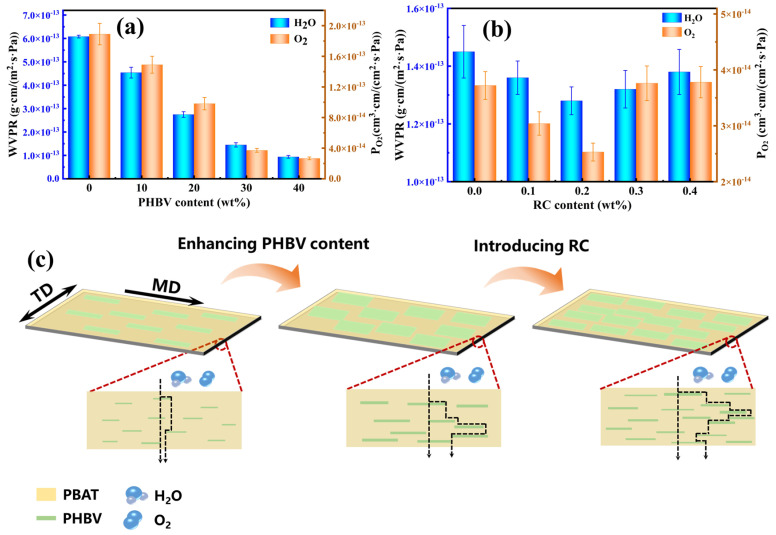
(**a**) WVTR and OTR of PBAT/PHBV films with the increase in PHBV content; (**b**) WVTR and OTR of PBAT/PHBV/RC films with the increase in RC content; (**c**) the schematic diagram of barrier pathway tortuosity influenced by lamellar structure.

The high barrier property represents a critical technical criterion for promoting degradable materials as substitutes for traditional packaging plastics, as it effectively extends food shelf life and maintains the soil moisture retention of agricultural mulch films. Figure 14 presents the water vapor and oxygen barrier properties of biodegradable blend films alongside the underlying mechanistic illustrations. Specifically, Figure 14a presents the evolution of water vapor transmission rate (WVTR) and oxygen transmission rate (OTR) for PBAT/PHBV films as a function of PHBV content. Upon increasing PHBV content to 40 wt.%, WVTR decreases from 6.1 × 10^−13^ to 9.0 × 10^−14^ g·cm/(m^2^·s·Pa), representing an 85% reduction, while OTR decreases from 1.9 × 10^−13^ to 2.7 × 10^−14^ cm^3^·cm/(cm^2^·s·Pa), demonstrating a comparable 85% decline. As a semi-crystalline polymer, PHBV inherently tends to form dense crystalline domains within the matrix. With increasing PHBV loading, the film crystallinity improves, where the crystalline phase acts as a physical barrier to disrupt the diffusion pathways of water vapor and oxygen molecules, thereby reducing permeability. The rigid molecular backbone of PHBV facilitates tighter packing of polymer chains at high loadings, which decreases free volume between chains and suppresses the migration of small molecules (e.g., H_2_O, O_2_) [46]. Notably, when PHBV sheet are arranged in parallel orientation within the PBAT matrix (as visualized in Figure 14c), the tortuosity of gas diffusion pathways is significantly enhanced, leading to the pronounced improvement in water vapor and oxygen barrier performance [47]. This finding also demonstrates that PBAT and PHBV exhibit a certain degree of compatibility, thereby preventing the formation of extensive voids at the interface that could otherwise serve as fast-track pathways to accelerate gas diffusion [48]. Thus, the continuous PBAT phase—with its inherently high gas diffusion rate—is more likely to serve as the primary pathway for rapid gas permeation.

The dimensions and density of barrier lamellae exert profound impacts on film barrier properties. Herein, the study investigates how RC affects compatibilization between PBAT and PHBV to regulate PHBV lamellar size and explores the corresponding effect on barrier performance. Figure 14b shows the evolution of WVTR and OTR for PBAT/PHBV/RC films as a function of RC loading. A distinct trend is observed: both WVTR and OTR decrease initially and then increase with increasing RC content, achieving optimal barrier performance at 0.2 wt.% RC. This phenomenon is rationalized by the Nielsen model, which postulates that the tortuosity of permeation pathways for water and oxygen molecules at fixed volume fraction is directly governed by the size and number of PHBV lamellae [25,26]. As previously discussed, increasing RC content reduces the dimensions of PHBV lamellae, which could potentially adversely affect barrier performance. Conversely, the concurrent increase in PHBV lamellar number with RC addition contributes to improved barrier properties. These opposing effects culminate in a maximum tortuosity of permeation pathways at 0.2 wt.% RC. Figure 14c illustrates the schematic diagram of how lamellar structural changes influence barrier pathway tortuosity. Additionally, the PBAT-B-PHBV copolymer formed by the reaction of RC with PBAT and PHBV tends to accumulate at the interfaces, acting as a compatibilizer. This enhances interfacial adhesion and reduces voids, which is beneficial for inhibiting gas diffusion [49].

## 4. Conclusions

This work established that in situ lamellar structuring via compatibilization and orientation control represents an effective approach to simultaneously enhance mechanical and barrier properties in fully degradable polymer systems. By introducing RC into PBAT/PHBV blends, branched/cross-linked PBAT-B-PHBV was formed to reduce domain size and improve interfacial adhesion. Biaxial stretching during blow-molding induced the transformation of PHBV sea–island structures into well-aligned lamellar architectures, creating a unique macroscopically single-layered/microscopically multilayered film architecture. The optimal RC content of 0.2 wt.% balanced lamellar density and lamellar size which strengthen permeation pathway tortuosity. Under a blow-up ratio of 4.5 and a draw ratio of 6.7, the PHBV phase in PBAT/PHBV/RC0.2 was transformed into oriented ribbon-like lamellae with an average length of 3.1 μm, width of 2.1 μm and thickness of 238 nm. Key results demonstrate that the independently oriented lamellae in the PBAT matrix led to a >600% increase in yield strength, >200% increase in elastic modulus and approximately 81%/85% reductions in water vapor/oxygen transmission rate, respectively. Besides, PHBV lamellae alignment enhances tear resistance, with optimal content varying by direction (30 wt.% MD, 20 wt.% TD), until excessive loading induces brittle fracture. The developed method offers a scalable pathway for producing high-performance biodegradable films, which can be readily applied in packaging materials, agricultural mulch films and other functional applications requiring tailored material properties.

## Figures and Tables

**Table 1:** The crystallization and melting parameters of PBAT/PHBV and PBAT/PHBV/RC blends.

Samples	T_g1_ (°C)	T_g2_ (°C)	T_c1_ (°C)	T_c2_ (°C)	T_c3_ (°C)	T_cc_ (°C)	ΔH_c1_ (J/g)	ΔH_c2_ (J/g)	ΔH_cc_ (J/g)	T_m1_ (°C)	T_m2_ (°C)	ΔH_m1_ (J/g)	ΔH_m2_ (J/g)
PBAT	−30.9	-	79.9	-	-	-	16.2	-	-	124.5	-	15.9	-
PBAT/PHBV10	−30.3	1.6	88.3	-	-	-	12.9	-	-	126.2	-	12.1	-
PBAT/PHBV20	−30.6	1.7	89.4	-	-	42.5	10.6	-	0.2	127.6	172.0	9.6	0.6
PBAT/PHBV30	−30.3	1.7	90.8	-	115.5	44.3	8.2	6.4	2.4	128.1	171.7	4.9	12.3
PBAT/PHBV40	−30.1	1.8	91.3	-	114.7	45.2	5.2	19.3	0.8	130.0	172.7	2.7	22.3
PHBV	-	1.7	-	-	126.6	-	-	86.7	-	-	174.5	-	86.9
PBAT/PHBV/RC0.1	30.4	1.7	87.4	-	115.4	44.4	8.4	5.8	3.5	126.9	171.9	4.9	12.7
PBAT/PHBV/RC0.2	30.7	1.8	87.5	-	115.8	44.8	8.4	3.5	3.2	126.6	171.9	4.6	10.8
PBAT/PHBV/RC0.3	30.6	1.7	85.6	109.0	115.5	45.1	8.3	3.0	2.7	126.4	171.4	4.5	10.0
PBAT/PHBV/RC0.4	30.7	1.6	86.2	109.5	115.7	44.3	7.9	3.9	2.0	126.6	170.8	4.6	10.1

## Data Availability

The original contributions presented in this study are included in the article. Further inquiries can be directed to the corresponding authors.
